# 2-((*E*)-{3-[(*E*)-2-Hy­droxy-3,5-diiodo­benzyl­idene­amino]-2,2-dimethyl­prop­yl}imino­meth­yl)-4,6-diiodo­phenol

**DOI:** 10.1107/S1600536812003704

**Published:** 2012-02-04

**Authors:** Hadi Kargar, Reza Kia, Tayebeh Shakarami, Muhammad Nawaz Tahir

**Affiliations:** a Department of Chemistry, Payame Noor University, PO Box 19395-3697 Tehran, I. R. of Iran; b X-ray Crystallography Laboratory, Plasma Physics Research Center, Science and Research Branch, Islamic Azad University, Tehran, Iran; c Department of Chemistry, Science and Research Branch, Islamic Azad University, Tehran, Iran; d Department of Physics, University of Sargodha, Punjab, Pakistan

## Abstract

The asymmetric unit of the title compound, C_19_H_18_I_4_N_2_O_2_, comprises a potentially tetra­dentate Schiff base ligand. The disordered H atoms on the N and O atoms were refined with site occupancies of 0.54 (8)/0.46 (8) and 0.59 (7)/0.41 (7), respectively. The dihedral angle between the benzene rings is 73.3 (3)°. Intra­molecular O—H⋯N and N—H⋯O hydrogen bonds make *S*(6) ring motifs. Short I⋯I [3.8919 (7) Å] and I⋯*Cg* [*Cg* is a ring centroid; 3.911 (2) Å] contacts are present in the crystal structure. The crystal structure is further stabilized by inter­molecular π–π inter­actions [centroid-to-centroid distance = 3.827 (3) Å].

## Related literature

For related structures, see for example: Kargar *et al.* (2011 ▶, 2012 ▶); Kia *et al.* (2010 ▶). For standard values of bond lengths, see: Allen *et al.* (1987 ▶). For details of hydrogen-bond motifs, see: Bernstein *et al.* (1995 ▶). For van der Waals radii, see: Bondi (1964 ▶).
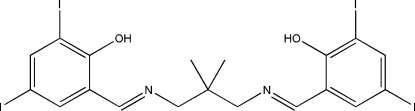


## Experimental

### 

#### Crystal data

C_19_H_18_I_4_N_2_O_2_
*M* *_r_* = 813.95
Orthorhombic, 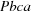

*a* = 12.2057 (5) Å
*b* = 11.8169 (5) Å
*c* = 31.8157 (15) Å
*V* = 4588.9 (3) Å^3^
*Z* = 8
Mo *K*α radiation
μ = 5.45 mm^−1^
*T* = 291 K
0.18 × 0.12 × 0.08 mm

#### Data collection

Bruker SMART APEXII CCD area-detector diffractometer
Absorption correction: multi-scan (*SADABS*; Bruker, 2005 ▶) *T*_min_ = 0.441, *T*_max_ = 0.670
39183 measured reflections
5472 independent reflections
2871 reflections with *I* > 2σ(*I*)
*R*_int_ = 0.099

#### Refinement

*R*[*F* ^2^ > 2σ(*F* ^2^)] = 0.044
*wR*(*F* ^2^) = 0.079
*S* = 1.00
5471 reflections
248 parameters
H-atom parameters constrained
Δρ_max_ = 1.11 e Å^−3^
Δρ_min_ = −0.94 e Å^−3^

### 

Data collection: *APEX2* (Bruker, 2005 ▶); cell refinement: *APEX2*; data reduction: *SAINT* (Bruker, 2005 ▶); program(s) used to solve structure: *SHELXTL* (Sheldrick, 2008 ▶); program(s) used to refine structure: *SHELXTL*; molecular graphics: *SHELXTL*; software used to prepare material for publication: *SHELXTL* and *PLATON* (Spek, 2009 ▶).

## Supplementary Material

Crystal structure: contains datablock(s) global, I. DOI: 10.1107/S1600536812003704/vm2150sup1.cif

Structure factors: contains datablock(s) I. DOI: 10.1107/S1600536812003704/vm2150Isup2.hkl

Supplementary material file. DOI: 10.1107/S1600536812003704/vm2150Isup3.cml

Additional supplementary materials:  crystallographic information; 3D view; checkCIF report

## Figures and Tables

**Table 1:** Hydrogen-bond geometry (Å, °)

*D*—H⋯*A*	*D*—H	H⋯*A*	*D*⋯*A*	*D*—H⋯*A*
N1—H1*A*⋯O1	0.72	1.90	2.526 (6)	145
O2—H2⋯N2	0.82	1.82	2.548 (6)	148

**Fig. 1.**
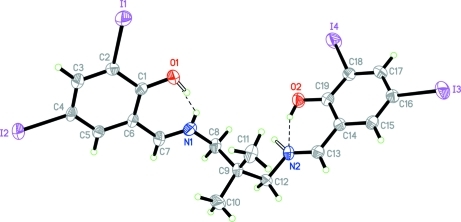
ORTEP plot of the title compound, showing 40% probability displacement ellipsoids and atomic numbering. The dashed lines show the intramolecular hydrogen bonds. The open bonds show the minor disordered components.

**Fig. 2.**
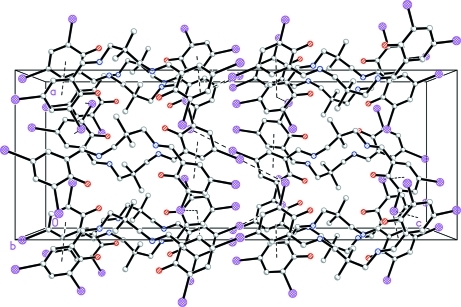
Packing diagram of the title compound viewed down the b-axis showing the short intermolecular I···I, I···Cg [C144–C19], and Cg [C1–C6]···Cg [C14–C19] contacts (dashed lines). All H atoms were omitted for clarity.

## supplementary crystallographic information

### Comment

In continuation of our work on the crystal structure of Schiff base ligands (Kargar *et al.*, 2011, 2012; Kia *et al.*, 2010), we determined the X-ray structure of the title compound.

The asymmetric unit of the title compound, Fig. 1, comprises a potentially tetradentate Schiff base ligand. In the crystal structure the phenolic H atoms are disordered over a second position on the Schiff base N atoms. The bond lengths and angles are within the normal ranges (Allen *et al.*, 1987) and are comparable to related structures (Kargar *et al.*, 2011, 2012; Kia *et al.*, 2010).

The intramolecular N—H···O and O—H···N hydrogen bonds (Table 1) make S(6) ring motifs (Bernstein *et al.*, 1995). The dihedral angle between the benzene rings is 73.3 (3)°. Short I(1)···I(2)^i^ [3.8919 (7) Å; (i) 1/2 + *x*, 1/2 - *y*, 1 - *z* ] and I4···*Cg2*^ii^ [3.911 (2) Å; (ii) 3/2 - *x*, -1/2 + *y*, *z*; *Cg*2 is the centroid of the C14–C19 ring] contacts are present in the crystal structure (Fig. 2). The crystal structure is further stabilized by the intermolecular π–π interaction [*Cg*1···*Cg*2^i^ = 3.827 (3)Å; (i) 2 - *x*, 1/2 + *y*, 1/2 - *z*; *Cg*1 and *Cg*2 are the centroids of the C1–C6 and C14–C19 benzene rings].

### Experimental

The title compound was synthesized by adding 3,5-diiodo-salicylaldehyde (2 mmol) to a solution of 2,2-dimethyl-1,3-propanediamine (1 mmol) in ethanol (30 ml). The mixture was refluxed with stirring for half an hour. The resultant solution was filtered. Yellow single crystals of the title compound suitable for X-ray structure determination were recrystallized from ethanol by slow evaporation of the solvents at room temperature over several days.

### Refinement

The O- and N-bound H atoms were located in difference Fourier map and then constrained to refine to the parent atoms with U_iso_(H) = 1.2U_eq_(N) and U_iso_(H) = 1.5U_eq_(O), respectively. The C-bound H-atoms were included in calculated positions and treated as riding atoms: C—H = 0.93, 0.96 and 0.97 Å for CH, CH_3_ and CH_2_ H-atoms, respectively, with U_iso_(H) = k x U_eq_(C), where k = 1.5 for CH_3_ H-atoms, and k = 1.2 for all other H-atoms.

### Crystal data

**Table:**

C_19_H_18_I_4_N_2_O_2_	*F*(000) = 2992
*M**_r_* = 813.95	*D*_x_ = 2.356 Mg m^−^^3^
Orthorhombic, *P**b**c**a*	Mo *K*α radiation, λ = 0.71073 Å
Hall symbol: -P 2ac 2ab	Cell parameters from 3211 reflections
*a* = 12.2057 (5) Å	θ = 2.5–27.5°
*b* = 11.8169 (5) Å	µ = 5.45 mm^−^^1^
*c* = 31.8157 (15) Å	*T* = 291 K
*V* = 4588.9 (3) Å^3^	Block, yellow
*Z* = 8	0.18 × 0.12 × 0.08 mm

### Data collection

**Table:**

Bruker SMART APEXII CCD area-detector diffractometer	5472 independent reflections
Radiation source: fine-focus sealed tube	2871 reflections with *I* > 2σ(*I*)
Graphite monochromator	*R*_int_ = 0.099
φ and ω scans	θ_max_ = 27.9°, θ_min_ = 1.3°
Absorption correction: multi-scan (*SADABS*; Bruker, 2005)	*h* = −15→16
*T*_min_ = 0.441, *T*_max_ = 0.670	*k* = −15→14
39183 measured reflections	*l* = −41→39

### Refinement

**Table:**

Refinement on *F*^2^	Primary atom site location: structure-invariant direct methods
Least-squares matrix: full	Secondary atom site location: difference Fourier map
*R*[*F*^2^ > 2σ(*F*^2^)] = 0.044	Hydrogen site location: inferred from neighbouring sites
*wR*(*F*^2^) = 0.079	H-atom parameters constrained
*S* = 1.00	*w* = 1/[σ^2^(*F*_o_^2^) + (0.0213*P*)^2^] where *P* = (*F*_o_^2^ + 2*F*_c_^2^)/3
5471 reflections	(Δ/σ)_max_ = 0.001
248 parameters	Δρ_max_ = 1.11 e Å^−^^3^
0 restraints	Δρ_min_ = −0.94 e Å^−^^3^

### Special details

**Table:**

Geometry. All esds (except the esd in the dihedral angle between two l.s. planes) are estimated using the full covariance matrix. The cell esds are taken into account individually in the estimation of esds in distances, angles and torsion angles; correlations between esds in cell parameters are only used when they are defined by crystal symmetry. An approximate (isotropic) treatment of cell esds is used for estimating esds involving l.s. planes.
Refinement. Refinement of F^2^ against ALL reflections. The weighted R-factor wR and goodness of fit S are based on F^2^, conventional R-factors R are based on F, with F set to zero for negative F^2^. The threshold expression of F^2^ > 2sigma(F^2^) is used only for calculating R-factors(gt) etc. and is not relevant to the choice of reflections for refinement. R-factors based on F^2^ are statistically about twice as large as those based on F, and R- factors based on ALL data will be even larger.

### Fractional atomic coordinates and isotropic or equivalent isotropic displacement parameters (Å^2^)

**Table:**

	*x*	*y*	*z*	*U*_iso_*/*U*_eq_	Occ. (<1)
I1	0.67018 (4)	0.26848 (5)	0.383838 (17)	0.06718 (18)	
I2	1.02767 (3)	0.17675 (4)	0.508635 (14)	0.04527 (13)	
I3	0.82622 (4)	0.00536 (4)	0.001078 (17)	0.06243 (17)	
I4	0.65930 (4)	−0.30987 (4)	0.131933 (17)	0.06028 (17)	
O1	0.8328 (3)	0.1220 (4)	0.32918 (15)	0.0501 (12)	
H1	0.8784	0.0964	0.3128	0.075*	0.46 (8)
O2	0.8775 (3)	−0.2231 (3)	0.17564 (15)	0.0475 (12)	
H2	0.9371	−0.2077	0.1860	0.071*	0.59 (7)
N1	0.9942 (4)	−0.0017 (4)	0.31029 (17)	0.0421 (14)	
H1A	0.9424	0.0253	0.3063	0.051*	0.54 (8)
N2	1.0582 (4)	−0.1182 (4)	0.18514 (18)	0.0421 (14)	
H1B	1.0104	−0.1580	0.2007	0.051*	0.41 (7)
C1	0.8751 (5)	0.1317 (5)	0.3665 (2)	0.0337 (15)	
C2	0.8201 (4)	0.1925 (5)	0.3985 (2)	0.0379 (16)	
C3	0.8633 (5)	0.2052 (5)	0.4382 (2)	0.0365 (16)	
H3	0.8259	0.2468	0.4584	0.044*	
C4	0.9642 (5)	0.1548 (5)	0.44789 (19)	0.0333 (15)	
C5	1.0178 (5)	0.0919 (5)	0.41839 (19)	0.0331 (15)	
H5	1.0838	0.0574	0.4254	0.040*	
C6	0.9755 (5)	0.0782 (5)	0.37794 (19)	0.0313 (14)	
C7	1.0307 (5)	0.0096 (5)	0.3477 (2)	0.0370 (16)	
H7	1.0947	−0.0278	0.3554	0.044*	
C8	1.0466 (5)	−0.0734 (5)	0.2792 (2)	0.0436 (17)	
H8A	0.9906	−0.1139	0.2637	0.052*	
H8B	1.0916	−0.1290	0.2935	0.052*	
C9	1.1191 (5)	−0.0066 (5)	0.2478 (2)	0.0381 (16)	
C10	1.2243 (5)	0.0305 (6)	0.2697 (2)	0.067 (2)	
H10A	1.2693	0.0719	0.2503	0.100*	
H10B	1.2634	−0.0350	0.2794	0.100*	
H10C	1.2065	0.0780	0.2932	0.100*	
C11	1.0597 (5)	0.0973 (5)	0.2311 (2)	0.054 (2)	
H11A	1.1027	0.1317	0.2093	0.081*	
H11B	1.0490	0.1505	0.2535	0.081*	
H11C	0.9898	0.0752	0.2198	0.081*	
C12	1.1517 (4)	−0.0858 (5)	0.2119 (2)	0.0426 (17)	
H12A	1.2066	−0.0488	0.1947	0.051*	
H12B	1.1844	−0.1537	0.2236	0.051*	
C13	1.0478 (5)	−0.0796 (5)	0.1485 (2)	0.0392 (17)	
H13	1.1029	−0.0336	0.1377	0.047*	
C14	0.9534 (5)	−0.1038 (5)	0.1222 (2)	0.0340 (15)	
C15	0.9413 (5)	−0.0561 (5)	0.0826 (2)	0.0359 (16)	
H15	0.9962	−0.0092	0.0723	0.043*	
C16	0.8510 (5)	−0.0761 (5)	0.0583 (2)	0.0361 (16)	
C17	0.7708 (5)	−0.1491 (5)	0.0727 (2)	0.0406 (17)	
H17	0.7100	−0.1646	0.0560	0.049*	
C18	0.7804 (5)	−0.1982 (5)	0.1111 (2)	0.0352 (16)	
C19	0.8699 (5)	−0.1780 (5)	0.1381 (2)	0.0329 (15)	

### Atomic displacement parameters (Å^2^)

**Table:**

	*U*^11^	*U*^22^	*U*^33^	*U*^12^	*U*^13^	*U*^23^
I1	0.0476 (3)	0.1081 (5)	0.0458 (3)	0.0294 (3)	0.0003 (3)	−0.0007 (3)
I2	0.0508 (3)	0.0558 (3)	0.0292 (2)	0.0012 (2)	−0.0041 (2)	−0.0020 (2)
I3	0.0643 (3)	0.0714 (4)	0.0516 (4)	0.0043 (2)	−0.0106 (3)	0.0231 (3)
I4	0.0551 (3)	0.0626 (3)	0.0631 (4)	−0.0240 (2)	−0.0120 (3)	0.0108 (3)
O1	0.041 (3)	0.078 (3)	0.031 (3)	0.007 (2)	−0.005 (2)	−0.010 (3)
O2	0.049 (3)	0.056 (3)	0.037 (3)	−0.012 (2)	−0.002 (2)	0.005 (2)
N1	0.038 (3)	0.053 (4)	0.035 (4)	−0.002 (3)	0.009 (3)	−0.007 (3)
N2	0.044 (3)	0.055 (4)	0.027 (4)	0.000 (3)	−0.002 (3)	−0.008 (3)
C1	0.030 (4)	0.043 (4)	0.028 (4)	−0.009 (3)	0.005 (3)	0.003 (3)
C2	0.029 (4)	0.052 (4)	0.033 (4)	−0.001 (3)	0.002 (3)	0.002 (3)
C3	0.044 (4)	0.034 (4)	0.032 (4)	−0.004 (3)	0.006 (3)	−0.004 (3)
C4	0.042 (4)	0.037 (4)	0.022 (4)	−0.006 (3)	0.006 (3)	0.005 (3)
C5	0.030 (3)	0.043 (4)	0.027 (4)	−0.002 (3)	0.004 (3)	0.007 (3)
C6	0.039 (4)	0.032 (4)	0.023 (4)	−0.001 (3)	0.006 (3)	0.000 (3)
C7	0.047 (4)	0.034 (4)	0.030 (4)	−0.002 (3)	0.004 (3)	−0.005 (3)
C8	0.047 (4)	0.053 (4)	0.031 (4)	−0.001 (3)	0.002 (3)	−0.009 (4)
C9	0.038 (4)	0.050 (4)	0.027 (4)	−0.002 (3)	−0.002 (3)	−0.009 (3)
C10	0.046 (5)	0.103 (7)	0.051 (6)	−0.016 (4)	−0.008 (4)	−0.033 (5)
C11	0.069 (5)	0.054 (5)	0.038 (5)	0.002 (4)	0.008 (4)	0.001 (4)
C12	0.030 (4)	0.066 (5)	0.032 (4)	−0.003 (3)	−0.002 (3)	−0.011 (4)
C13	0.036 (4)	0.043 (4)	0.038 (5)	0.003 (3)	0.003 (3)	−0.016 (4)
C14	0.034 (4)	0.038 (4)	0.030 (4)	−0.002 (3)	−0.002 (3)	−0.003 (3)
C15	0.040 (4)	0.032 (4)	0.036 (4)	0.001 (3)	0.005 (3)	−0.001 (3)
C16	0.039 (4)	0.034 (4)	0.035 (4)	0.010 (3)	−0.002 (3)	0.000 (3)
C17	0.035 (4)	0.044 (4)	0.043 (5)	0.000 (3)	−0.009 (3)	−0.002 (4)
C18	0.037 (4)	0.031 (4)	0.038 (4)	−0.007 (3)	0.000 (3)	−0.005 (3)
C19	0.040 (4)	0.024 (4)	0.034 (4)	0.002 (3)	0.005 (3)	−0.004 (3)

### Geometric parameters (Å, º)

**Table:**

I1—C2	2.091 (6)	C8—C9	1.550 (8)
I2—C4	2.098 (6)	C8—H8A	0.9700
I3—C16	2.081 (6)	C8—H8B	0.9700
I4—C18	2.090 (6)	C9—C11	1.522 (8)
O1—C1	1.301 (7)	C9—C10	1.526 (8)
O1—H1	0.8203	C9—C12	1.530 (8)
O2—C19	1.312 (7)	C10—H10A	0.9600
O2—H2	0.8201	C10—H10B	0.9600
N1—C7	1.278 (8)	C10—H10C	0.9600
N1—C8	1.452 (7)	C11—H11A	0.9600
N1—H1A	0.7184	C11—H11B	0.9600
N2—C13	1.259 (8)	C11—H11C	0.9600
N2—C12	1.474 (7)	C12—H12A	0.9700
N2—H1B	0.8984	C12—H12B	0.9700
C1—C2	1.416 (8)	C13—C14	1.452 (8)
C1—C6	1.426 (8)	C13—H13	0.9300
C2—C3	1.376 (8)	C14—C15	1.388 (8)
C3—C4	1.403 (8)	C14—C19	1.436 (8)
C3—H3	0.9300	C15—C16	1.367 (8)
C4—C5	1.364 (8)	C15—H15	0.9300
C5—C6	1.396 (8)	C16—C17	1.383 (8)
C5—H5	0.9300	C17—C18	1.359 (8)
C6—C7	1.427 (8)	C17—H17	0.9300
C7—H7	0.9300	C18—C19	1.408 (8)
			
C1—O1—H1	110.0	C12—C9—C8	108.6 (5)
C19—O2—H2	109.8	C9—C10—H10A	109.5
C7—N1—C8	122.8 (6)	C9—C10—H10B	109.5
C7—N1—H1A	115.1	H10A—C10—H10B	109.5
C8—N1—H1A	121.8	C9—C10—H10C	109.5
C13—N2—C12	121.2 (6)	H10A—C10—H10C	109.5
C13—N2—H1B	129.4	H10B—C10—H10C	109.5
C12—N2—H1B	108.7	C9—C11—H11A	109.5
O1—C1—C2	120.8 (6)	C9—C11—H11B	109.5
O1—C1—C6	122.4 (6)	H11A—C11—H11B	109.5
C2—C1—C6	116.7 (6)	C9—C11—H11C	109.5
C3—C2—C1	122.2 (6)	H11A—C11—H11C	109.5
C3—C2—I1	119.6 (5)	H11B—C11—H11C	109.5
C1—C2—I1	118.2 (5)	N2—C12—C9	112.8 (5)
C2—C3—C4	119.5 (6)	N2—C12—H12A	109.0
C2—C3—H3	120.2	C9—C12—H12A	109.0
C4—C3—H3	120.2	N2—C12—H12B	109.0
C5—C4—C3	120.0 (6)	C9—C12—H12B	109.0
C5—C4—I2	121.6 (5)	H12A—C12—H12B	107.8
C3—C4—I2	118.4 (4)	N2—C13—C14	122.8 (6)
C4—C5—C6	121.3 (6)	N2—C13—H13	118.6
C4—C5—H5	119.3	C14—C13—H13	118.6
C6—C5—H5	119.3	C15—C14—C19	119.4 (6)
C5—C6—C1	120.1 (5)	C15—C14—C13	121.8 (6)
C5—C6—C7	120.9 (6)	C19—C14—C13	118.8 (6)
C1—C6—C7	119.0 (6)	C16—C15—C14	122.0 (6)
N1—C7—C6	121.5 (6)	C16—C15—H15	119.0
N1—C7—H7	119.2	C14—C15—H15	119.0
C6—C7—H7	119.2	C15—C16—C17	119.4 (6)
N1—C8—C9	113.2 (5)	C15—C16—I3	122.2 (5)
N1—C8—H8A	108.9	C17—C16—I3	118.4 (5)
C9—C8—H8A	108.9	C18—C17—C16	120.2 (6)
N1—C8—H8B	108.9	C18—C17—H17	119.9
C9—C8—H8B	108.9	C16—C17—H17	119.9
H8A—C8—H8B	107.8	C17—C18—C19	122.9 (6)
C11—C9—C10	109.2 (5)	C17—C18—I4	119.6 (5)
C11—C9—C12	110.9 (5)	C19—C18—I4	117.6 (5)
C10—C9—C12	107.3 (5)	O2—C19—C18	122.7 (6)
C11—C9—C8	111.4 (5)	O2—C19—C14	121.2 (6)
C10—C9—C8	109.4 (6)	C18—C19—C14	116.1 (6)

### Hydrogen-bond geometry (Å, º)

**Table:**

*D*—H···*A*	*D*—H	H···*A*	*D*···*A*	*D*—H···*A*
N1—H1*A*···O1	0.72	1.90	2.526 (6)	145
O2—H2···N2	0.82	1.82	2.548 (6)	148

## References

[bb1] Allen, F. H., Kennard, O., Watson, D. G., Brammer, L., Orpen, A. G. & Taylor, R. (1987). *J. Chem. Soc. Perkin Trans. 2*, pp. S1–19.

[bb2] Bernstein, J., Davis, R. E., Shimoni, L. & Chang, N.-L. (1995). *Angew. Chem. Int. Ed. Engl.* **34**, 1555–1573.

[bb3] Bondi, A. (1964). *J. Phys. Chem.* **68**, 441–452.

[bb4] Bruker (2005). *APEX2*, *SAINT* and *SADABS* Bruker AXS Inc., Madison, Wisconsin, USA.

[bb5] Kargar, H., Kia, R., Abbasian, S. & Tahir, M. N. (2012). *Acta Cryst.* **E**68, o142. 10.1107/S1600536811053438 PMC3254485 22259428

[bb6] Kargar, H., Kia, R., Pahlavani, E. & Tahir, M. N. (2011). *Acta Cryst.* **E**67, o614. 10.1107/S1600536811004776 PMC3052006 21522371

[bb7] Kia, R., Kargar, H., Tahir, M. N. & Kianoosh, F. (2010). *Acta Cryst.* **E**66, o2296. 10.1107/S1600536810031430 PMC3008013 21588648

[bb8] Sheldrick, G. M. (2008). *Acta Cryst.* A**64**, 112–122. 10.1107/S0108767307043930 18156677

[bb9] Spek, A. L. (2009). *Acta Cryst.* D**65**, 148–155. 10.1107/S090744490804362X PMC2631630 19171970

